# AaeAP2a, a scorpion-derived antimicrobial peptide, combats carbapenem-resistant *Acinetobacter baumannii* via membrane disruption and triggered metabolic collapse

**DOI:** 10.3389/fmicb.2025.1673333

**Published:** 2025-10-28

**Authors:** Weiyu Luo, Liuwei Zhang, Haolei Gao, Huiying Li, Xiaofeng Li, Yuliang Wen, Huarun Sun, Bolin Hang, Longfei Zhang, Wei Zhang, Xuehan Liu, Ruibiao Wang, Bo Wen, Jiyuan Shen, Chunling Zhu, Yueyu Bai, Lei Wang, Ke Ding, Jianhe Hu

**Affiliations:** ^1^College of Animal Science and Veterinary Medicine, Henan Institute of Science and Technology, Xinxiang, China; ^2^Postdoctoral Research Base, Henan Institute of Science and Technology, Xinxiang, China; ^3^Postdoctoral Research Base, College of Veterinary Medicine, Henan Agricultural University, Zhengzhou, China; ^4^Ministry of Education Key Laboratory for Animal Pathogens and Biosafety, Zhengzhou, China; ^5^Laboratory of Functional Microbiology and Animal Health, Henan University of Science and Technology, Luoyang, China

**Keywords:** Crab, antimicrobial peptide, AaeAP2a, bactericidal mechanism, peritonitis-associated sepsis

## Abstract

**Introduction:**

Carbapenem-resistant *Acinetobacter baumannii* (CRAB) poses a significant global health challenge owing to its high mortality rates and widespread antibiotic resistance. While the clinical utility of last-resort antibiotics, such as colistin, remains limited. Consequently, developing novel antimicrobial agents is imperative. Antimicrobial peptides have emerged as promising candidates against multidrug-resistant pathogens. Animal venom constitutes a rich reservoir of bioactive peptides.

**Methods:**

In this study, in vitro experiments were conducted to assess the antibacterial activity of the scorpion-derived peptide AaeAP2a against CRAB, its inhibition of biofilm formation, as well as its stability and biocompatibility. Additionally, the antibacterial mechanism was investigated, and in vivo efficacy was evaluated using a mouse model of peritonitis-associated sepsis.

**Results:**

AaeAP2a exhibits potent antibacterial activity against CRAB and a significant inhibitory effect on biofilm formation. Moreover, AaeAP2a maintains high stability under a broad range of stressful physicochemical conditions and exhibits promising biocompatibility *in vitro*. Mechanistically, AaeAP2a disrupts bacterial membrane integrity, increases membrane permeability, reduces the NAD^+^/NADH ratio, dissipates the proton motive force, decreases ATP production, and induces reactive oxygen species and hydroxyl radical accumulation. Moreover, in a mouse model of peritonitis-associated sepsis, AaeAP2a treatment enhanced survival rates and reduced bacterial burdens in key organs.

**Discussion:**

These findings underscore the potential of AaeAP2a as a promising therapeutic agent for CRAB infections, offering novel strategies for addressing antimicrobial resistance.

## 1 Introduction

The escalating antibiotic resistance crisis has extended beyond the healthcare sector, posing a systemic threat within the One Health framework (Aslam et al., 2021; de la Rocque et al., 2023). The World Health Organization (WHO) named carbapenem-resistant *Acinetobacter baumannii* (CRAB) as the pathogen with the highest alert status in 2017 (Yadav et al., 2025). The emergence of CRAB has further heightened morbidity and mortality rates related to *A. baumannii* infections, with these rates ranging from 33 to 86% (Monteiro et al., 2025). Notably, studies have observed that the resistance rate of *Acinetobacter baumannii* to carbapenems surged from 4.7 to 62.8% between 2016 and 2020 (Ju et al., 2023). This alarming status is attributable not only to the overuse of antibiotics but also to the rapid dissemination of drug-resistant genes across various hosts (e.g., humans, companion animals) and environmental settings (e.g., healthcare facilities, pet hospitals, soil, and water ecosystems) (Castañeda-Barba et al., 2024). According to growing data, companion animals, due to their shared close-contact environments with humans (such as households and veterinary clinics), facilitate the exchange of drug-resistant bacteria and genes, thus serving as key vectors for the transmission of antimicrobial resistance (AMR) (Zhao et al., 2022; Price et al., 2024). Such cross-species transmission complicates traditional infection control measures and underscores the necessity of shifting from a “human-centered” approach to a holistic One Health perspective. Colistin is the current last-line defense antibiotic for CRAB; nonetheless, in recent years, there has been a growing number of reports on colistin-resistant *Acinetobacter baumannii* (Liu et al., 2022). Moreover, its clinical utility is limited due to its nephrotoxicity and neurotoxicity (Guzman et al., 2025a, 2025b). Additionally, colistin overuse has led to the emergence of the mcr-1 resistance gene, first identified in 2015 and spread through plasmids. This accelerates colistin resistance and further jeopardizes its clinical effectiveness (Dalmasso et al., 2023). In light of these challenges, there is an urgent scientific imperative to develop safe and effective alternative antimicrobial agents.

Due to their unique mechanisms of action and broad-spectrum efficacy (Bucataru and Ciobanasu, 2024), antimicrobial peptides (AMPs), which are essential components of the innate immune system, are a promising treatment for multidrug-resistant (MDR) infections (Gao et al., 2024). In addition to its primary neurotoxins, scorpion venom also contains a wide range of bioactive molecules, including AMPs that exhibit antibacterial, antiviral, immunosuppressive, analgesic, and anticancer effects (Rabea et al., 2025; Xin et al., 2025). This study introduces AaeAP2a, an optimized analog of the naturally derived AaeAP2, originally isolated from scorpion venom. Although AaeAP2 exhibits broad-spectrum antimicrobial activity, its efficacy against Gram-negative bacteria is limited (MIC for *Escherichia coli* >512 μg/mL). Conversely, the modified and synthesized analog AaeAP2a significantly reduces the MIC for *E. coli* to 16 μg/mL and demonstrates enhanced inhibitory effects on the proliferation of cancer cells, including lung (H460), breast (MB435S, MCF-7), and prostate (PC-3) cancer cell lines (Du et al., 2015). Nevertheless, the antibacterial activity and underlying mechanisms of AaeAP2a against MDR Gram-negative pathogens, particularly CRAB, remain insufficiently characterized.

The objective of this study was to investigate the antimicrobial activity and mechanisms of AaeAP2a against CRAB. Using a CRAB strain isolated from a pet dog as the target pathogen, we observed that AaeAP2a exhibited significant antibacterial activity and dose-dependent inhibition of biofilm formation. Moreover, AaeAP2a demonstrated excellent stability, promising biocompatibility *in vitro*, and a low propensity for inducing resistance. Mechanistically, AaeAP2a disrupted both inner and outer bacterial membranes, increased membrane permeability, dissipated membrane potential, inhibited ATP synthesis, and induced oxidative stress, cumulatively leading to bacterial death. Notably, AaeAP2a also proved effective in a mouse model of peritonitis-associated sepsis.

## 2 Materials and methods

### 2.1 Antimicrobial peptide

The antimicrobial peptide AaeAP2a (FLFKLIPKAIKGLVKAIRK; molecular weight: 2183.84 Da) was synthesized by GL Biochemistry (Shanghai, China) via solid-phase chemical synthesis. The purity of the peptide (>95%) was confirmed by reversed-phase high-performance liquid chromatography (RP-HPLC), and its sequence was validated using electrospray ionization mass spectrometry (ESI-MS).

### 2.2 Bacterial strains and gene detection

The *A. baumannii* strain ATCC 19606 was obtained from the American Type Culture Collection (ATCC). The CRAB 236 antibiotic-resistant clinical strain maintained in our laboratory was originally isolated from a pet dog. Polymerase chain reaction (PCR) was used to amplify resistance genes, including *bla_OXA-23_*, *bla_OXA-24_*, *bla_OXA-51_*, and *bla_OXA-58_*. Additionally, the virulence genes *bap*, *bfs*, *bfmS*, *adeH*, *csuA*, *csuAB*, *pgaA*, *abaI*, *basD*, and *ompA* were detected (Table 1).

**Table 1:** PCR primer sequences and amplicon lengths of target genes.

Target gene	Primer sequence (5′–3′)	Length (bp)
*bla_OXA-23_*	*F: CAGAATATGTGCCAGCCTCT	534
	*R: ATTTCTGACCGCATTTCCAT	
*bla_OXA-24_*	*F: GGTTAGTTGGCCCCCTTAAA	249
	*R: AGTTGAGCGAAAAGGGGATT	
*bla_OXA-51_*	*F: TAATGCTTTGATCGGCCTTG	353
	*R: TGGATTGCACTTCATCTTGG	
*bla_OXA-58_*	*F: AAGTATTGGGGCTTGTGCTG	599
	*R: CCCCTCTGCGCTCTACATAC	
*bap*	*F: TCATCAGAATTCCAAGGTGT	267
	*R: CTAACCATTCAGCTTCAG	
*bfs*	*F: GCGCATATGAAAAATGATGC	453
	*R: GCGCTCGAGTCATTTCAAAT	
*bfmS*	*F: CCAAGACCAAAGTGTCATGT	788
	*R: TATGAATACCGCCCGTAATC	
*adeH*	*F: CAACTGAATGAACTTGAACAG	281
	*R: GCTGCGTTGACACTACTTGC	
*csuA*	*F: ATGATATTCAATCGTGGTTC	549
	*R: TTAAAACTCAATCGTAATTG	
*csuAB*	*F: ATGAAAAACATTCAGAAATC	514
	*R: GAGTATCTTTATAATCGCCT	
*pgaA*	*F: ATTGCTGCAAGCGAGCGAAA	276
	*R: ACCGCTTTTGAACGCCCTCT	
*abaI*	*F: GTACAGTCGACGTATTTGTTG	382
	*R: CGTACGTCTAGAGTAATGAGTT	
*basD*	*F: CTCTCCCCAAAGAATGGATC	847
	*R: AGCGCCATGATGACTGAGTT	
*ompA*	*F: CTGCTCCATTAGCTGCTGCT	560
	*R: AGGCTTCAAGTGACCACCAAG	

*F and R indicate the forward and reverse primers, respectively.

### 2.3 Evaluation of inhibitory effect (minimum inhibitory concentration, MIC)

The inhibitory activity of the antimicrobial peptide was assessed using the broth microdilution method following the CLSI M100-ED34 standard (Humphries et al., 2021). A single colony of *A. baumannii* was isolated and inoculated into Mueller-Hinton Broth (MHB), then incubated overnight at 37 °C with agitation at 180 rpm. The overnight cultures were adjusted to a 0.5 McFarland turbidity standard, after which it was diluted 1:100 in MHB medium. The MICs of tetracycline, ciprofloxacin, gentamicin, ceftriaxone, colistin, meropenem, and the antimicrobial peptide AaeAP2a for the CRAB 236 strain were subsequently determined using the broth microdilution method. *A. baumannii* ATCC 19606 served as the control strain, and the experiments were conducted in triplicate. The Mueller-Hinton Broth medium and the antibiotics used (tetracycline, ciprofloxacin, gentamicin, ceftriaxone, colistin, meropenem) were all purchased from Macklin (China).

### 2.4 Time-kill curve

The bactericidal potential of AaeAP2a was evaluated using time-kill curve assays on *A. baumannii* strains ATCC 19606 and CRAB 236 (Yi et al., 2022). After the bacterial cultures were diluted in MHB at a 1:100 ratio, the AaeAP2a was applied in final concentrations corresponding to 0.5, 1, 2, and 4 × MIC, with a control group without the AMPs. Samples were analyzed using 10-fold serial dilutions at 0, 2, 4, 6, 8, 10, and 12 h, and 100 μL aliquots were placed on Mueller-Hinton Agar (MHA) plates. The plates were incubated at 37 °C for 24 h before colony enumeration. Triplicate assays were performed for each condition. Mueller-Hinton Agar (MHA) medium was purchased from Macklin (China); and the incubator was from Thermo Fisher Scientific (USA).

### 2.5 Determination of biofilm inhibitory activity

In this study, the crystal violet staining method was employed to evaluate the inhibitory efficacy of the antimicrobial peptide AaeAP2a on *A. baumannii* biofilm formation. An *A. baumannii* overnight culture was diluted in MHB to yield a bacterial suspension of 1 × 10^7^ CFU/mL; this suspension was prepared from cultures incubated at 37 °C with agitation at 180 rpm. Subsequently, 150 μL of the suspension was dispensed into each well of a sterile 96-well plate. AaeAP2a was added at final concentrations ranging from 0.5 × to 4 × the MIC. A negative control group, which received no AMP, was established, while colistin at 1 × MIC served as the positive control. After incubation at 37 °C for 24 h, the supernatant was removed, and the remaining biofilm was subjected to staining with crystal violet, followed by washing, and then solubilization. The absorbance was then measured at 570 nm to quantify the biofilm mass (Grasteau et al., 2011). All experiments were performed in triplicate. All reagents used in this experiment were obtained from Macklin (China). The incubator and microplate reader were from Thermo Fisher Scientific (United States); and the sterile 96-well microplates were purchased from Corning (United States).

### 2.6 Stability analysis

We thoroughly examined the effects of storage at room temperature, repeated freeze–thaw cycles, temperature variations, pH levels, salt ions, urea, and protease treatment on the antimicrobial activity of peptide AaeAP2a (Zhu et al., 2022). Through 0 to 12 freeze–thaw iterations, AaeAP2a was incubated for 1 h at various temperatures (0, 25, 50, 75, and 100 °C) and pH values (3 to 11). Additionally, the peptide was incubated with four salt ions—NaCl, KCl, CaCl_2_, and MgCl_2_—at concentrations of 0, 10, 50, 100, 150, and 200 mmol/mL, as well as with urea at concentrations of 0, 2, 4, 6, 8, and 10 mmol/mL for 1 h. Furthermore, AaeAP2a was treated with proteinase K or trypsin at concentrations ranging from 0 to 100 μg/mL (i.e., 0, 20, 40, 60, 80, and 100 μg/mL) for 1 h at 37 °C. The changes in its antimicrobial activity were quantitatively assessed using the MIC method. All assays were conducted in triplicate. Notably, for this section, only the highly representative standard strain ATCC 19606 was specifically selected as the experimental strain. Proteinase K, Trypsin, and Urea were obtained from Beyotime Biotechnology (China), while all other reagents were purchased from Macklin (China).

### 2.7 Biosafety assay

To systematically evaluate the biosafety of the antimicrobial peptide AaeAP2a, we assessed its hemolytic activity and cytotoxicity (Liu et al., 2020a; Zhou F. et al., 2022). A 4% (v/v) erythrocyte suspension was prepared from freshly collected anticoagulated mouse blood by repeatedly resuspending and washing the cells three times with phosphate-buffered saline (PBS), following the established protocol. This suspension was incubated with a series of AaeAP2a portions (0, 2, 4, 8, 16, 32, and 64 × MIC) at 37 °C for 1 h. In this test, 1% (v/v) Triton X-100 was used as the positive control. Three replicates per treatment group were used to study OD at 540 nm using a microplate reader. The cytotoxicity of AaeAP2a on human embryonic kidney 293 T cells was tested using the Cell Counting Kit - 8 (CCK-8). In this experiment, different AaeAP2a concentrations (0, 0.5, 1, 2, 4, 8, 16, and 32 MIC) were added to DMEM-containing wells containing 1 × 10^4^ cells/well and 10% (v/v) fetal bovine serum, followed by 24-h incubation at 37 °C. The CCK-8 assay was thus performed, with three replicates recorded for each concentration.

### 2.8 Assessment of resistance development

*A. baumannii* ATCC 19606 in the exponential growth phase was inoculated into MHB supplemented with a sub-inhibitory concentration of AaeAP2a (0.5 × MIC). Colistin served as the positive control at a 0.5 × MIC, while an untreated group was included as the negative control. Following incubation at 37 °C with shaking at 180 rpm for 24 h, the MIC for each group was determined. This incubation and MIC determination procedure was repeated 20 times, after which the fold change in MIC relative to the initial baseline was calculated (Pollard et al., 2012). Sources of key reagents used in the experiment are as follows: Phosphate-Buffered Saline (PBS), 1% Triton X-100 solution, and Cell Counting Kit-8 (CCK-8) were all purchased from Beyotime Biotechnology (China); Dulbecco’s Modified Eagle Medium (DMEM) and Fetal Bovine Serum (FBS) were both sourced from Gibco (United States).

### 2.9 Outer membrane permeability assay

Bacterial cells from an overnight culture of activated *A. baumannii* were collected by centrifugation, washed twice with 5 mM HEPES, and resuspended to an OD_600_ of 0.5 (approximately 1.5 × 10^8^ CFU/mL). The fluorescent probe N-phenylnaphthylamine (Merck; 1-N-phenyl-naphthylamine, NPN) was then added to a final concentration of 10 μM, and the mixture was incubated at 37 °C for 30 min (Zhu et al., 2022). Subsequently, varying concentrations (0.5–4 × MIC) of AaeAP2a or colistin (1 × MIC) were introduced, and the samples were further incubated for 1 h. The fluorescence intensity was measured at an excitation wavelength of 350 nm and an emission wavelength of 420 nm.

### 2.10 Assessment of cytoplasmic membrane integrity

A cell suspension was prepared as described in section 2.9, and propidium iodide (PI) was added to a final concentration of 0.5 μM. AaeAP2a was administered at concentrations ranging from 0.5 to 4 × MIC, with colistin (1 × MIC) as a positive control after the initial incubation at 37 °C for 30 min. After an additional hour of incubation, a microplate reader with excitation and emission frequencies of 535 and 615 nm was used to determine the light strength (Zhu et al., 2022). Propidium Iodide (PI) and colistin were purchased from Macklin (China).

### 2.11 Scanning electron microscopy analysis

Scanning Electron Microscopy (SEM) was used to evaluate the effects of the antimicrobial peptide AaeAP2a on the morphology of *A. baumannii* cells. Overnight-cultured *A. baumannii* cells were washed with PBS and resuspended to an OD_600_ of 0.5. AaeAP2a was added to a final concentration of 4 × MIC, and the samples were incubated at 37 °C for 2 and 4 h, respectively. Following incubation, the samples were fixed in a 2.5% glutaraldehyde solution at 4 °C overnight. The fixed samples were subsequently dehydrated using a graded ethanol series (30, 50, 70, 90, and 100%), dried, sputter-coated with a conductive metal, and finally examined by SEM. SEM analysis was performed with a chamber vacuum of ≤2 × 10^−4^ Pa, an acceleration voltage of 3.00 kV, a probe current of 100 pA, and a working distance of 7.00 mm. The 2.5% glutaraldehyde fixative was obtained from Solarbio (China); and scanning electron microscopy (SEM) was performed using a ZEISS Sigma 300 instrument from Oberkochen, Germany.

### 2.12 Determination of cell membrane potential

The bacterial suspension was prepared as described in section 2.9, and 3,3’-Dipropylthiadicarbocyanine iodide [DiSC3(5)] was added to a final concentration of 0.5 μM, and the mixture was incubated at 37 °C for 30 min. Subsequently, AaeAP2a (0.5–4 × MIC) and colistin (at 1 × MIC, serving as the positive control) were added, whereas an untreated control group was maintained without drug administration. The bacterial suspension was incubated at 37 °C for an additional hour, and the fluorescence intensity was measured using a microplate reader at excitation/emission wavelengths of 622/670 nm (Dombach et al., 2020). The DiSC3(5) probe was obtained from Thermo Fisher Scientific (United States).

### 2.13 Determination of cell pH gradient

A bacterial suspension was prepared as described in Section 2.9, and the pH-sensitive fluorescent probe 2′,7′-bis(carboxyethyl)-5(6)-carboxyfluorescein acetate methyl ester (BCECF-AM, 20 μM) was added for incubation. After that, AaeAP2a (0.5–4 × MIC) and colistin (1 × MIC) were introduced, and the cells were kept at 37 °C for 1 h. Each sample was analyzed sequentially using a microplate reader with 500 nm and 522 nm excitation and emission wavelengths. The pH-sensitive fluorescent probe BCECF-AM was purchased from Invitrogen (Thermo Fisher Scientific, United States).

### 2.14 ATP assay

According to the reference’s instructions (Yi et al., 2022), an ATP assay kit measured intracellular ATP levels in *A. baumannii* cells (Beyotime Biotechnology, China). Bacterial cultures were rinsed with PBS (pH 7.4) and adjusted to an OD_600_ of 0.5. The cells were then exposed to AaeAP2a (0.5–4 × MIC) or colistin (1 × MIC) for 1 h. Following incubation, the supernatant was removed after centrifugation, and the bacterial pellet was lysed using the ATP experiment lysis buffer. The resulting supernatant was subsequently used to identify intracellular ATP levels by a microplate reader.

### 2.15 Determination of total NAD^+^/NADH content

As previously described, the total NAD+/NADH content in *A. baumannii* was determined using the Beyotime NAD+/NADH Assay Kit based on the WST − 8 process (Li et al., 2019). Bacterial cultures, adjusted to an OD600 of 0.5, were incubated with various concentrations (0.5–4 × MIC) of AaeAP2a or colistin (1 × MIC) for 1 h. After centrifugation, the supernatant was removed, and the bacterial pellet was lysed using lysozyme. The lysate was soon centrifuged to obtain the supernatant, which was then incubated in the dark. At 450 nm, the absorbance was measured using a microplate reader. The NAD+/NADH Assay Kit (WST-8 method) was obtained from Beyotime Biotechnology (China); lysozyme was purchased from Sangon Biotech (China).

### 2.16 Determination of ROS content

Log-phase *A. baumannii* cultures adjusted to an OD_600_ of 0.5 were incubated with 10 μM 2′,7′-dichlorodihydrofluorescein diacetate (DCFH-DA) for 30 min, followed by treatment with varying concentrations (0.5–4 × MIC) of AaeAP2a or colistin (1 × MIC) for 1 h. Fluorescence strength was determined using a microplate reader at excitation and emission wavelengths of 488 nm and 525 nm, respectively (Liu et al., 2020b). The Reactive Oxygen Species Assay Kit containing the DCFH-DA fluorescent probe was obtained from Beyotime Biotechnology (China).

### 2.17 Measurement of hydroxyl radicals (•OH)

In the logarithmic growth phase, bacteria were washed and adjusted to an OD_600_ of 0.5 before 30 min of incubation with the hydroxyphenyl fluorescein (HPF) probe. The cells were then exposed to a range of AaeAP2a (0.5–4 × MIC) or colistin (1 × MIC, serving as a positive control). The treated cells were kept in the dark for an additional hour. The fluorescence intensity at 495 and 515, both at excitation and emission wavelengths, was determined using a microplate reader (Sugimoto et al., 2021). The fluorescent probe hydroxyphenyl fluorescein (HPF) and colistin were purchased from Macklin (China).

### 2.18 *In vivo* efficacy evaluation of AaeAP2a

To evaluate the antimicrobial efficacy of AaeAP2a *in vivo*, specific pathogen-free (SPF) grade BALB/c mice, aged 6 to 8 weeks and weighing 20 ± 2 g, with an equal sex distribution, were used to establish a mouse peritonitis-associated sepsis model. The mice were given 6.75 × 10^6^ CFU of CRAB 236 via intraperitoneal injection. Animals were randomly assigned to five groups (*n* = 10 per group): a PBS control group, a CRAB 236-infected group, and three treatment groups receiving low (5 mg/kg), medium (10 mg/kg), or high (20 mg/kg) doses of AaeAP2a. Treatment was administered via oral gavage at 2, 24, and 48 h post-infection, and the survival rate was monitored over 72 h. SPF-level BALB/c mice were obtained from Vital River Laboratory Animal Technology Co., Ltd. (China).

In a subsequent experiment under similar conditions, mice were administered AaeAP2a by gavage 2 h post-infection. Twelve hours post-drug administration, the mice were euthanized via cervical dislocation. Blood, liver, and spleen tissues were harvested and homogenized in sterile PBS, and serial dilutions were prepared. An aliquot of 0.1 mL from each dilution was evenly spread on LB agar plates, and bacterial counts in the target tissues were determined after overnight incubation. Each dilution was plated in triplicate. It is important to highlight that in our study, no protective measures (e.g., encapsulation or chemical modification) were applied to AaeAP2a for enhancing its stability. Oral gavage needles were acquired from Servicebio (China), and a tissue homogenizer was procured from Ningbo Scientz Biotechnology Co., Ltd. (SCIENTZ, China).

### 2.19 Data analysis

Statistical analyses were performed using GraphPad Prism version 9.5.0. Data, derived from a minimum of three biological replicates, are presented as the mean ± standard deviation (SD). Intergroup comparisons were conducted using a nonparametric one-way analysis of variance (ANOVA), with statistical significance defined as follows: *p < 0.05, **p < 0.01, ***p < 0.001, and ****p < 0.0001.

## 3 Results

### 3.1 CRAB 236 exhibits resistance and virulence-associated genes

**Figure 1:**
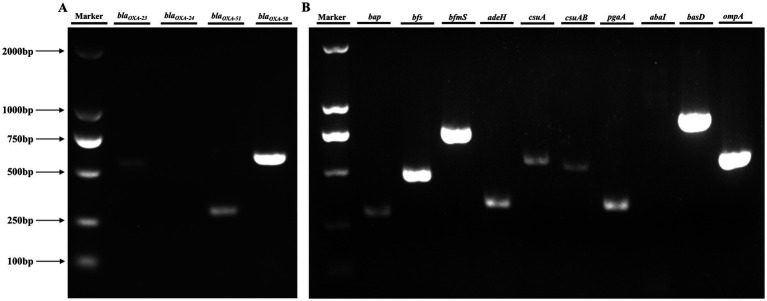
Analysis of resistance and virulence genes in the CRAB 236 strain. **(A)** Resistance gene detection demonstrated that CRAB 236 successfully amplified *bla_OXA-23_* (534 bp), *bla_OXA-51_* (353 bp), and *bla_OXA-58_* (599 bp). **(B)** Analysis of virulence genes showed that CRAB 236 tested positive for *bap, bfs, bfmS, adeH, csuA, csuAB, pgaA, basD*, and *ompA*. Lane Marker corresponds to the DL2000 DNA marker.

PCR amplification and subsequent gel electrophoresis were employed to screen for key genes associated with clinically significant traits in *A. baumannii*. Analysis revealed that CRAB 236 carried the carbapenem-resistance determinants *bla_OXA-23_*, *bla_OXA-51_*, and *bla_OXA-58_* (Figure 1A), which collectively encode OXA-type carbapenemases, the primary molecular mediators of resistance to carbapenems (Lupo et al., 2017). Virulence gene amplification confirmed that CRAB 236 carries *bap*, *bfs*, *bfmS*, *adeH*, *csuA*, *csuAB*, *pgaA*, *basD*, and *ompA* (Figure 1B), which mediate diverse pathogenic functions including biofilm formation, adhesion, and iron acquisition (Lukovic et al., 2025; Ali et al., 2025). These findings indicate that the strain exhibits a high level of carbapenem resistance, possesses robust biofilm-forming capabilities, and demonstrates significant pathogenic potential.

### 3.2 AaeAP2a exhibits complete bactericidal activity against CRAB

The MIC assay results indicated that isolate CRAB 236 exhibited resistance to a range of antibiotics, including tetracyclines, quinolones, aminoglycosides, and carbapenems (CLSI), thereby confirming its MDR profile. Notably, AaeAP2a demonstrated significant inhibitory activity against this resistant strain (MIC = 6.25 μg/mL) and exhibited even greater efficacy against the sensitive reference strain ATCC 19606 (MIC = 3.125 μg/mL) (Table 2). These findings suggest that AaeAP2a may overcome conventional antibiotic resistance mechanisms.

**Table 2:** Results of the MIC (μg/mL) assay.

Drugs		CRAB 236		ATCC 19606	
		MIC	Interpretation	MIC	Interpretation
	Tetracycline	64	R	2	S
	Ciprofloxacin	32	R	0.25	S
Antibiotic	Gentamicin	>32	R	1	S
	Ceftriaxone	32	I	16	I
	Colistin	0.5	S	2	S
	Meropenem	16	R	2	S
AMPs	AaeAP2a	6.25	–	3.125	–

R, Resistant; I, Intermediate; S, Sensitive. The symbol “–” indicates that standardized resistance criteria for antimicrobial peptides are currently unavailable.

**Figure 2:**
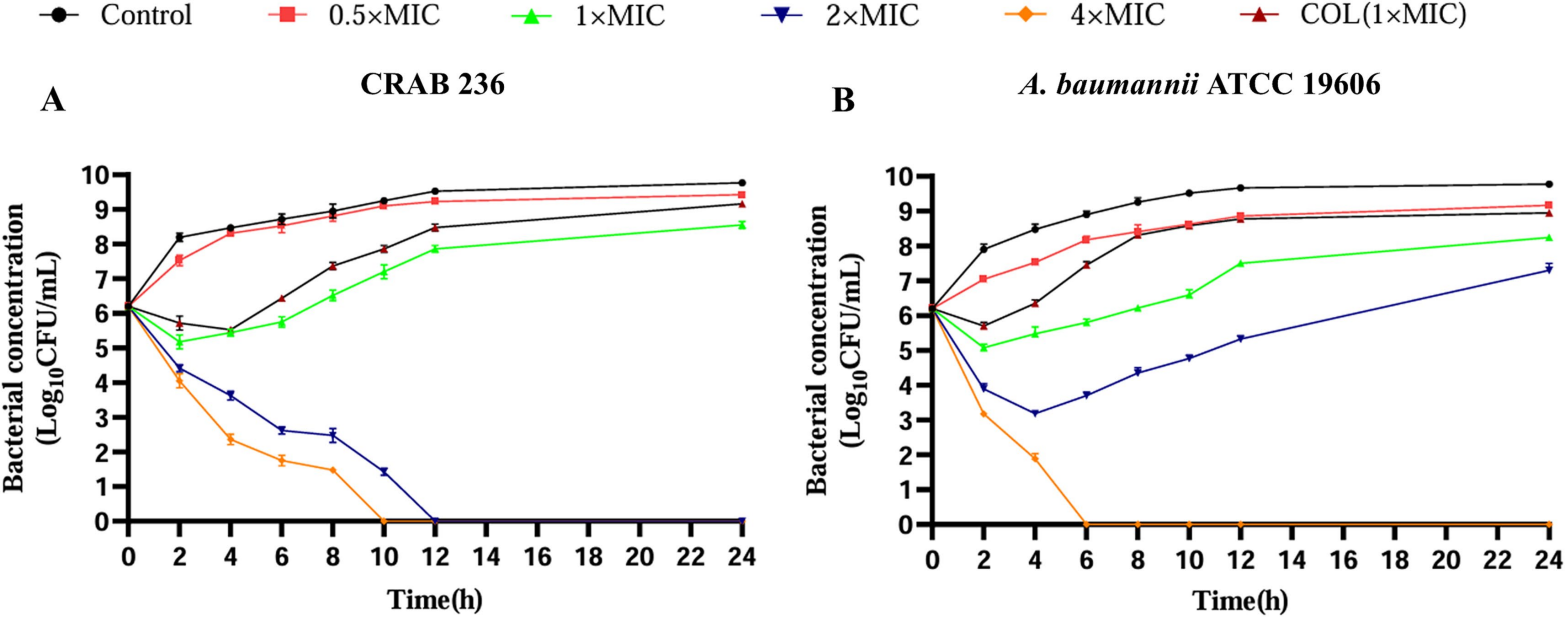
AaeAP2a demonstrates complete bactericidal activity against CRAB. The time-dependent bactericidal effects of AaeAP2a on strains CRAB 236 **(A)** and ATCC 19606 **(B)**, respectively. Data are presented as the mean ± SD (*n* = 3).

Further assessment of the dynamic bactericidal efficacy of AaeAP2a was performed using a time-kill curve analysis. AaeAP2a exhibited a potent, concentration-dependent bactericidal effect. Specifically, treatment with 4 × MIC (25 μg/mL) of AaeAP2a for 10 or 6 h resulted in complete eradication of the clinical isolate (CRAB 236) and the standard strain (ATCC 19606), respectively. Furthermore, treatment at a lower concentration of 2 × MIC fully eradicated the strain ATCC 19606 within 12 h (Figures 2A,B). These findings underscore the promise of AaeAP2a as a potential bactericidal antibiotic candidate against CRAB.

### 3.3 AaeAP2a exhibits low propensity for inducing drug resistance and effectively inhibits biofilm formation *in vitro*

**Figure 3:**
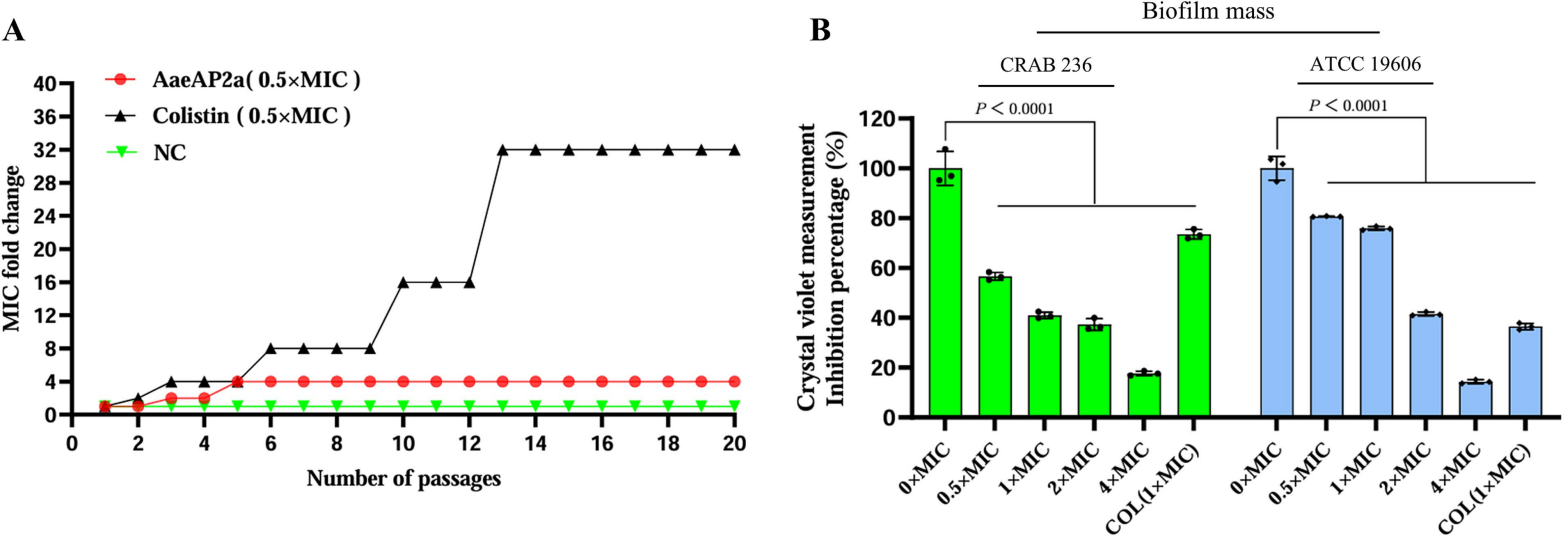
AaeAP2a exhibits low susceptibility to resistance and effectively inhibits biofilm formation. **(A)** In the resistance development experiment, the MIC of the AaeAP2a group (red) increased 4-fold over 20 generations, compared to a 32-fold increase in the colistin group (black), the negative control group (green) exhibited no changes. These values reflect the multiplicative change in MIC relative to the initial generation. **(B)** In the biofilm inhibition assay, AaeAP2a significantly reduced biofilm absorbance across concentrations ranging from 0.5 × MIC to 4 × MIC, achieving an inhibition rate exceeding 80% for both strains at 4 × MIC.

To assess the propensity of AaeAP2a to induce bacterial resistance, *A. baumannii* ATCC 19606 was repeatedly exposed to 0.5 × MIC (1.5625 μg/mL) of AaeAP2a over 20 consecutive days through serial passaging. The results showed a 4-fold increase in the AaeAP2a MIC (Figure 3A), which is considerably lower than the 32-fold increase observed with the colistin control (0.5 × MIC, 2 μg/mL). Meanwhile, the MIC of the negative control exhibited no significant changes, which thereby rules out the interference of “MIC alterations caused by spontaneous mutations of the strain itself.” This comparison indicates a minimal risk of resistance development with AaeAP2a, supporting its potential for long-term clinical application.

Additionally, bacterial biofilm formation significantly enhances pathogenicity and contributes to drug resistance, leading to chronic infections and treatment challenges (Vishwakarma et al., 2021; Ma et al., 2024; Olanrewaju et al., 2024). Notably, AaeAP2a was found to effectively inhibit biofilm formation in a concentration-dependent manner. As demonstrated in Figure 3B, treatment with AaeAP2a resulted in an 85% reduction in biofilm absorbance for ATCC 19606 and an 82% reduction for CRAB 236 at 4 × MIC (relative to the 0 × MIC group, *p* < 0.001). Notably, at an identical concentration of 1 × MIC, AaeAP2a demonstrated enhanced biofilm inhibitory activity against CRAB 236 compared to colistin. These findings suggest that AaeAP2a not only exhibits potent antibacterial activity but also diminishes bacterial pathogenicity by inhibiting biofilm formation, thereby underscoring its potential clinical utility.

### 3.4 AaeAP2a exhibits promising biocompatibility and stability *in vitro*

**Figure 4:**
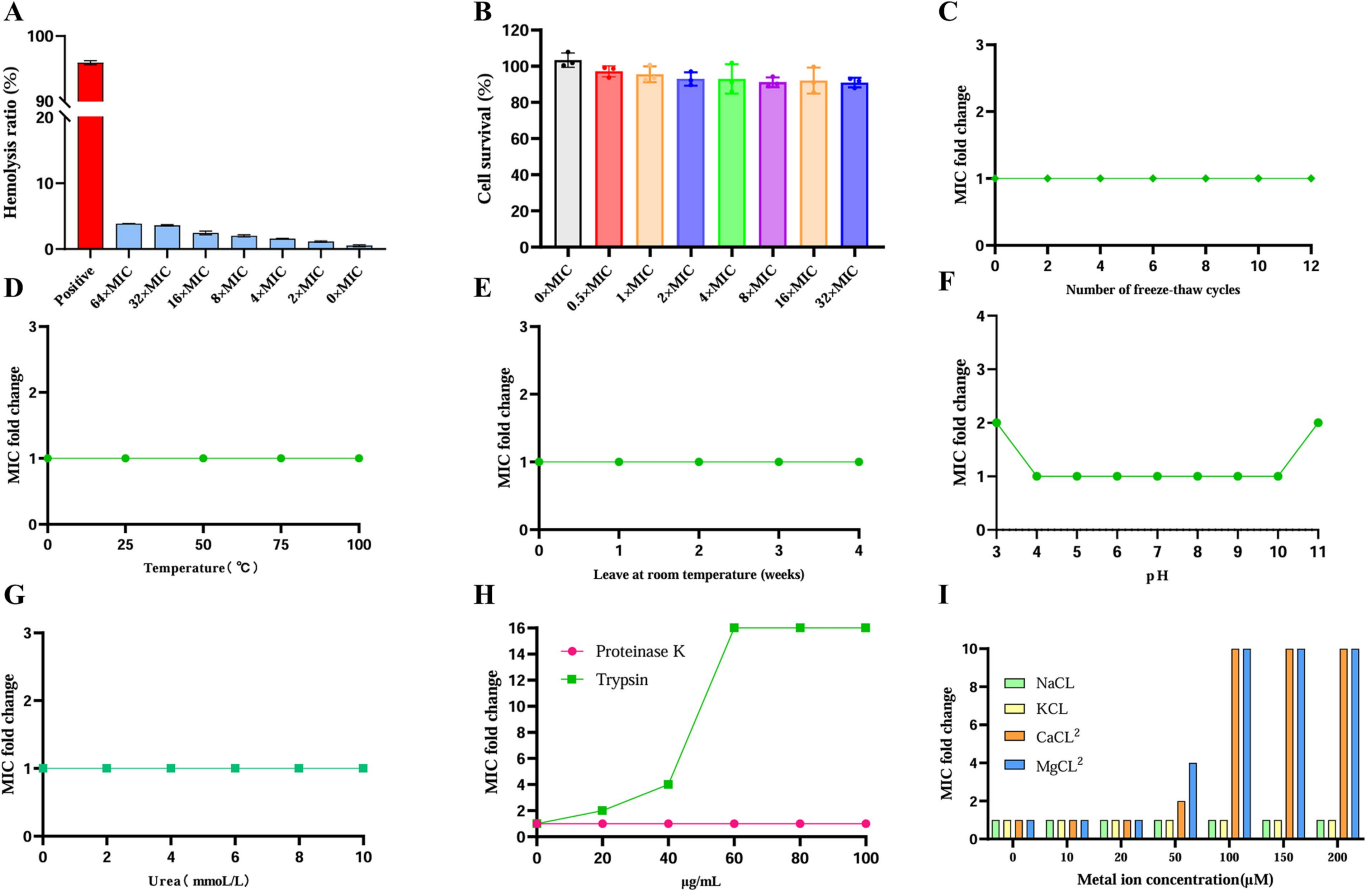
AaeAP2a demonstrates favorable biosafety and stability. **(A)** Hemolysis of mouse erythrocytes. **(B)** Cytotoxicity in 293 T cells. **(C)** Stability after freeze–thaw cycles (−80 to 25 °C). **(D)** Thermal stability at different temperatures. **(E)** Long-term stability during room temperature storage (25°C). **(F)** Stability across a range of pH values. **(G)** Urea tolerance. **(H)** Protease resistance. **(I)** Effect of metal ions.

Ensuring the safety and stability of a pharmaceutical agent is essential for its clinical application. In this study, we thoroughly evaluated the hemolytic activity, cytotoxicity, and overall stability of AaeAP2a under a range of conditions. At 16 × MIC, AaeAP2a induced < 5% hemolysis in mouse erythrocytes and maintained > 90% viability in 293 T cells. Even at 64 × MIC (200 μg/mL), hemolysis remained below 5% (Figures 4A,B). Moreover, regarding physical stability, AaeAP2a maintained full activity after undergoing 12 freeze–thaw cycles (from −80 to 25 °C), exposure to 100 °C for 1 h, or incubation across a pH range of 4–10 for 1 h (Figures 4C,D,F). Even under extreme pH conditions (pH 3 and pH 11), the MIC increased only twofold. Notably, its antibacterial efficacy remained undiminished after storage at room temperature for 4 weeks (Figure 4E). Concerning chemical stability, AaeAP2a demonstrated substantial resistance to urea and proteinase K, although its activity was markedly reduced at trypsin concentrations exceeding 40 μg/mL (Figures 4G,H). While the peptide was not affected by the presence of monovalent cations (Na^+^/K^+^), a 10-fold increase in the MIC was observed with 100 μM Ca^2+^ or Mg^2+^ (Figure 4I).

### 3.5 AaeAP2a disrupts the integrity of inner and outer membranes

**Figure 5:**
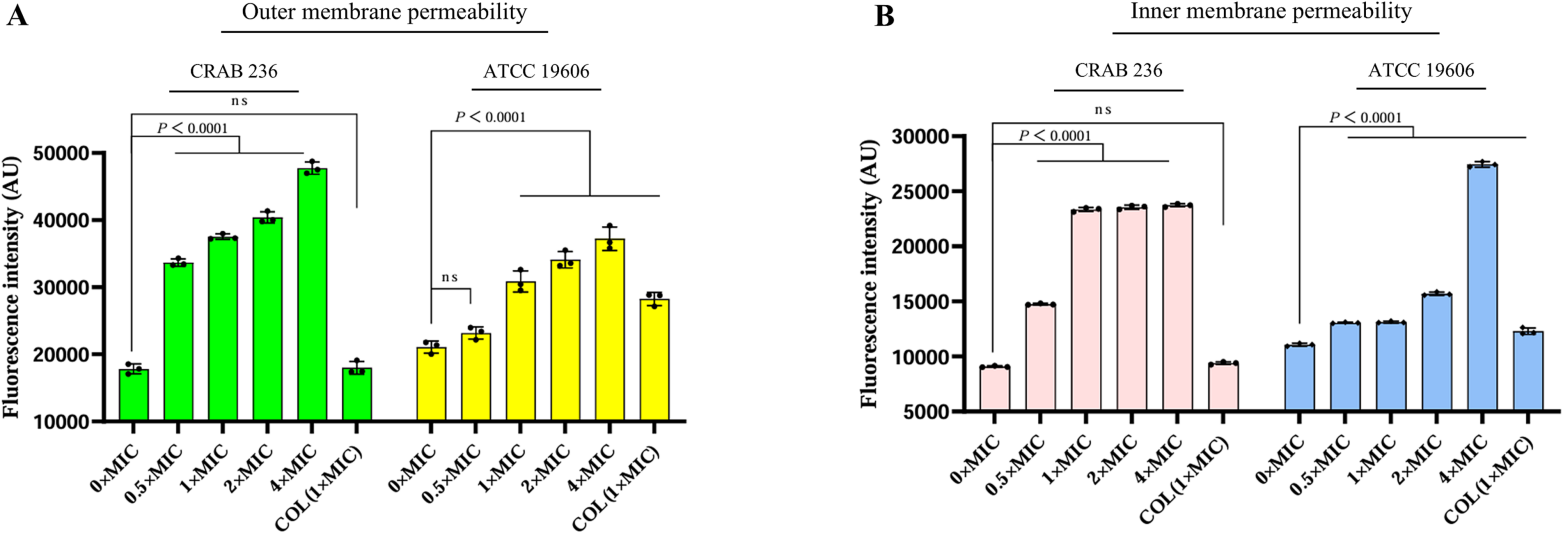
AaeAP2a influences the permeability of the outer and inner membranes of *A. baumannii*. **(A,B)** The assessment of membrane permeability after co-incubating AaeAP2a, at concentrations ranging from 0 to 4 × MIC, with either strain ATCC 19606 or CRAB 236 for 1 h. The assays employed 10 μM NPN or 0.5 μM PI as fluorescent indicators. All experiments were conducted in triplicate, with error bars representing the standard deviation (SD). Statistical significance was evaluated using a nonparametric one-way analysis of variance (ANOVA), and “AU” denotes arbitrary units.

**Figure 6:**
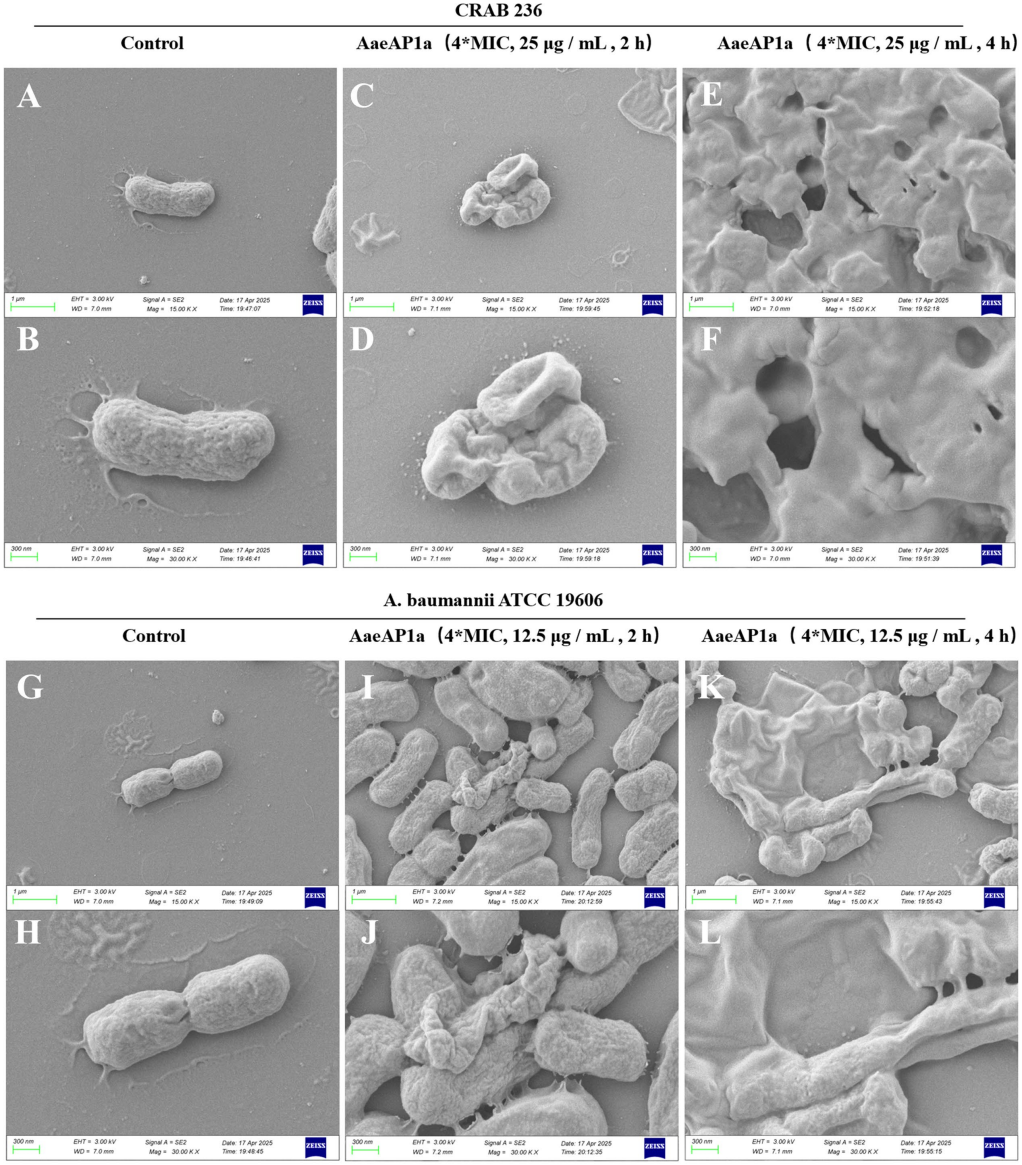
AaeAP2a disrupts the structure of the bacterial membrane. **(A–F)** SEM images of *A. baumannii* CRAB 236 post-treatment with 4 × MIC AaeAP2a (25 μg/mL) for either 2 or 4 h. **(G–L)** SEM images of *A. baumannii* ATCC 19606 after exposure to 4 × MIC AaeAP2a (12.5 μg/mL) for the same time intervals.

To elucidate the bactericidal mechanism of AaeAP2a, we evaluated its impact on the membrane structure of *A. baumannii* using fluorescent probe techniques and SEM. Fluorescence tracer experiments using 1-N-phenylnaphthylamine (NPN), an outer membrane probe, and propidium iodide (PI), an inner membrane probe, demonstrated that AaeAP2a significantly increased the permeability of both bacterial membranes in a dose-dependent manner. Specifically, CRAB 236 treated with a 0.5 × MIC of AaeAP2a exhibited greater fluorescence intensity for both NPN and PI compared with colistin at 1 × MIC (Figures 5A,B). At 1 × MIC, treatment with AaeAP2a resulted in a 2.3-fold increase in PI fluorescence intensity compared to colistin in the canine-derived CRAB 236 isolate (Figure 5B), indicating marked disruption of both membranes. These findings were further corroborated by SEM observations (Figure 6). After 2 h of treatment with a 4 × MIC of AaeAP2a, the bacterial membranes exhibited severe wrinkling and structural damage, with the deterioration becoming more pronounced over time and resulting in cytoplasmic leakage at 4 h. Consistent effects were observed in the reference strain ATCC 19606 (Figures 6G–L), mirroring the response seen in CRAB 236 (Figures 6A–F).

### 3.6 Multiple mechanisms synergistically enhance the anti-CRAB activity of AaeAP2a

**Figure 7:**
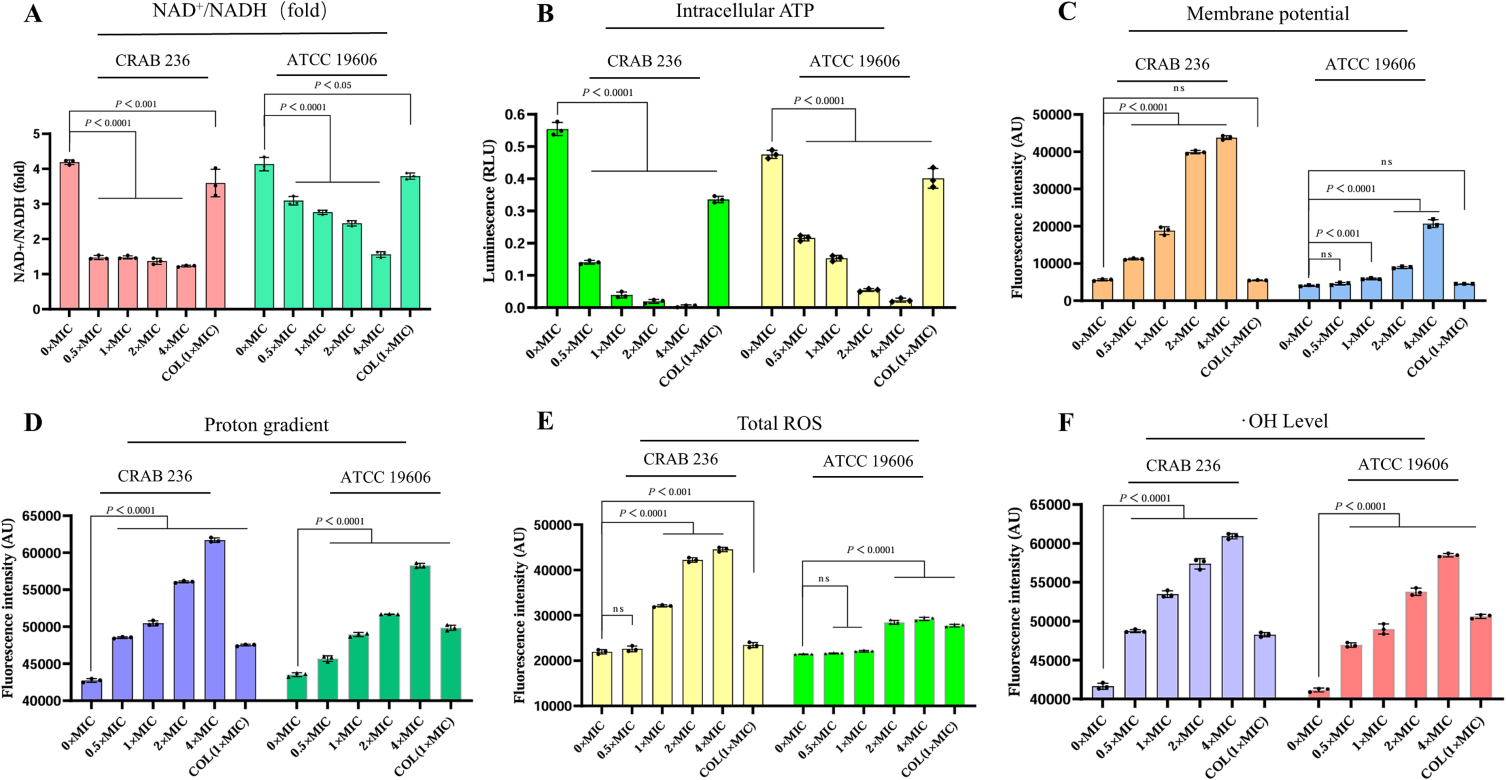
AaeAP2a modulates the NAD^+^/NADH ratio, disrupts the PMF, reduces ATP levels, and induces oxidative stress damage. **(A)** NAD^+^/NADH ratio. **(B)** Intracellular ATP levels. **(C)** Δψ measured using DiSC3(5). **(D)** ΔpH assessed with BCECF-AM. **(E)** ROS levels. **(F)** Hydroxyl radical accumulation. All experiments were conducted in triplicate, and the data are presented as mean ± standard deviation (mean ± SD).

Building on its excellent membrane permeability, we further elucidated the mechanism by which AaeAP2a synergistically disrupts the metabolic homeostasis of *A. baumannii* through multiple pathways. The NAD^+^/NADH ratio, an indicator of cellular energy metabolism and redox balance (Xu et al., 2023), was significantly reduced in a dose-dependent manner following treatment with AaeAP2a (Figure 7A). The bacterial PMF, which comprises an electrical potential (Δψ) and a transmembrane proton gradient (ΔpH), is an essential energy mechanism embedded in bacterial membranes. It is crucial for several physiological processes, including ATP creation and the movement of active substances. Because of its significance for bacterial survival, the PMF is a promising target for antimicrobial treatments (Yang et al., 2023). We assessed the impact of AaeAP2a on Δψ and ΔpH using DiSC3(5) and BCECF-AM, respectively. The results demonstrated that AaeAP2a induced dose-dependent membrane depolarization and potential dissipation (Figure 7C), alongside an elevation in intramembrane alkalinization (Figure 7D). Our assays revealed a significant drop in intracellular ATP levels (Figure 7B), which is consistent with the dissipation of PMF and the decrease in NAD+/NADH ratio, ultimately causing a collapse in energy metabolism. Additional investigations showed a rapid accumulation of reactive oxygen species (ROS) and hydroxyl radicals (•OH) (Figures 7E,F), which resulted in multiple oxidative stress-induced injuries.

### 3.7 AaeAP2a exhibits significant antimicrobial activity *in vivo*

**Figure 8:**
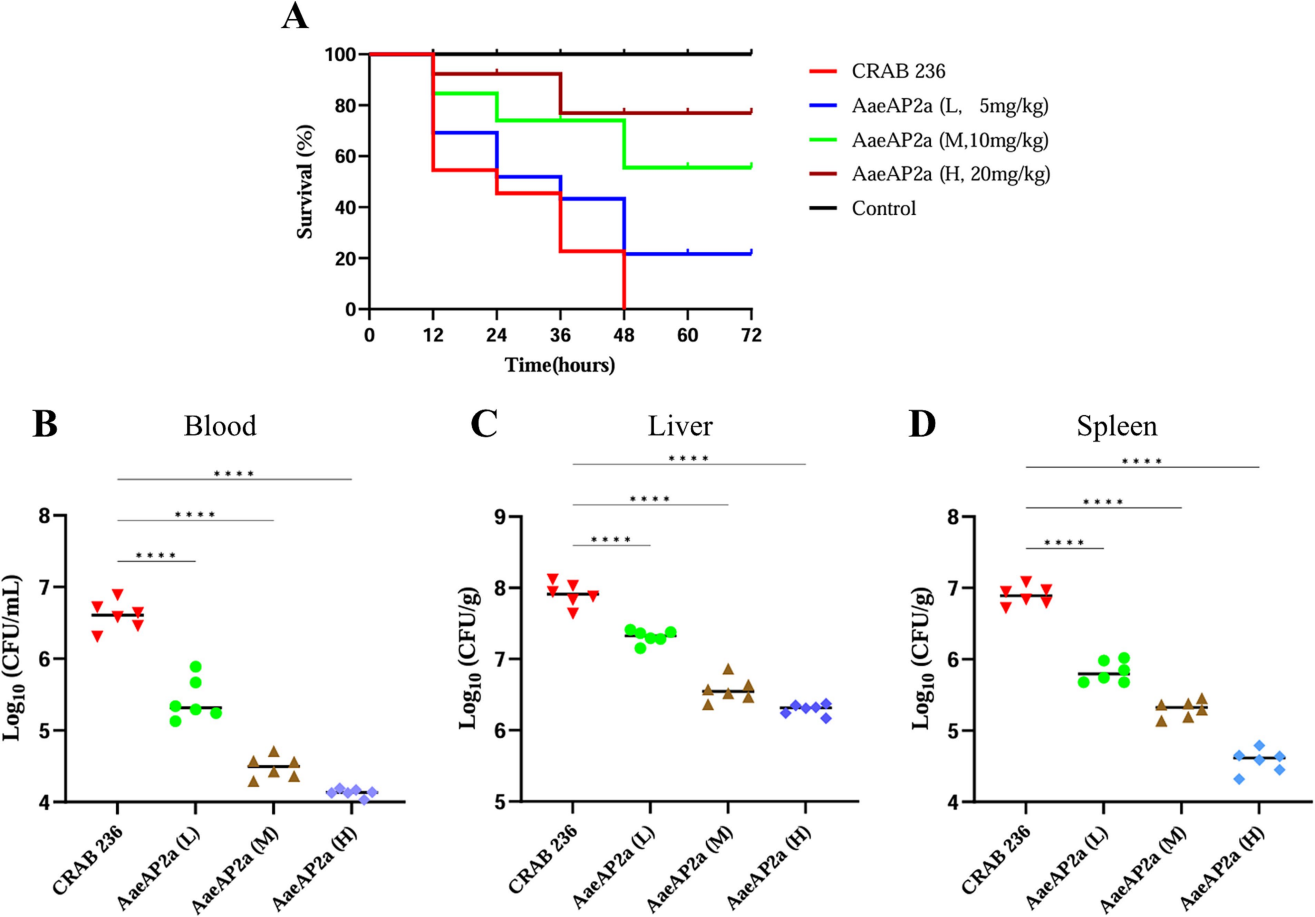
AaeAP2a demonstrates dose-dependent antimicrobial efficacy in a murine peritonitis-associated sepsis model. **(A)** Survival curves of infected mice (*n* = 10) treated with various doses of AaeAP2a (5–20 mg/kg) compared to the PBS control group. **(B–D)** Bacterial loads in the blood, liver, and spleen of infected mice 12 h post-administration of different doses of AaeAP2a. The symbol “****” indicates a statistically significant difference with a *P*-value less than 0.0001, meaning there is an extremely significant statistical difference between the relevant groups.

To evaluate the *in vivo* therapeutic efficacy of AaeAP2a, a peritonitis-associated sepsis model was established in BALB/c mice infected with CRAB 236 at a dose of 6.75 × 10^6^ CFU. The results demonstrated that all mice treated with PBS alone succumbed within 48 h post-infection. In contrast, increasing the dosage of AaeAP2a from 5 to 20 mg/kg led to a marked enhancement in the 72-h survival rate, rising from 20 to 80% (Figure 8A). Moreover, AaeAP2a significantly reduced bacterial loads in the blood, liver, and spleen in a dose-dependent manner 12 h after administration (Figures 8B–D), a finding that correlated with reduced symptom severity and improved survival.

## 4 Discussion

The evolutionary complexity of CRAB undermines conventional therapies through rapid resistance evolution, leading to high mortality rates among infected patients—particularly in cases of bloodstream infections (Luo et al., 2025), COVID-19-related complications (Russo et al., 2022), and post-craniotomy infections (Lan et al., 2024). Recent epidemiological investigations have revealed a concerning trend: companion animals have emerged as potential reservoirs for MDR pathogens, thereby facilitating the establishment of a human–animal–environment transmission network that poses an ongoing threat to public health and safety (Lupo et al., 2017). The co-occurrence of resistance, biofilm-associated, and virulence genes in the canine-derived CRAB 236 strain demonstrates carbapenem resistance, robust biofilm-forming capacity, and significant pathogenic potential. These findings are corroborated by phenotypic evidence in Table 2 (MIC assays), Figure 3B (biofilm quantification), and Figure 8A (murine virulence model), respectively. Based on these findings, we hypothesize that the CRAB 236 strain effectively overcomes host defenses and antimicrobial barriers through these polygenic co-evolutionary mechanisms. Consequently, traditional antibiotics relying on single-target mechanisms face increasing limitations in treating CRAB infections. Moreover, last-resort antibiotics like colistin remain clinically suboptimal due to constraints such as nephrotoxicity concerns (Guzman et al., 2025a, 2025b). Thus, developing innovative approaches to overcome multidrug resistance constraints is imperative.

The scientific community has recognized antimicrobial peptides—natural immune molecules that synergistically eradicate bacteria through multiple mechanisms and are less likely to induce resistance—as a promising solution against MDR bacteria (Song et al., 2021). Scorpion venoms are a powerful natural reservoir of AMPs, offering a foundation for the development of novel agents against a broad spectrum of pathogens, including MDR microbes, fungi, viruses, parasites, and malignant cells (Rabea et al., 2025). In this study, we systematically evaluated the antimicrobial activity, stability, security, and protection against CRAB of AaeAP2a. We also elucidated its antimicrobial mechanism and assessed its therapeutic efficacy *in vivo*.

In addition to increasing the permeability of antimicrobial agents, bacterial biofilms, which rely on a complex matrix of extracellular polymeric substances, support the evolution of bacterial drug resistance, creating a protective barrier against drug-induced killing (Verderosa et al., 2019). AMPs have shown dual potential in meeting biofilm-related chronic infections: they can inhibit the initial stages of biofilm formation and eradicate mature biofilms. In this study, AaeAP2a, at a 4 × MIC, obtained an inhibition rate approaching 80% against biofilms formed by two strains of *Acinetobacter baumannii* (Figure 3B). Consistent with previous findings, these results verify the high efficacy of AMPs in preventing biofilm formation (Jung et al., 2021; Wang et al., 2022). However, the recalcitrance of mature biofilms is the main obstacle to treating chronic infections. To tackle this clinical dilemma, several studies have shown that SR25, at a concentration of 1 × MIC, accomplishes a 56% eradication efficiency against *Escherichia coli* biofilms, profoundly surpassing colistin (35%) (Luo et al., 2024). Moreover, the helical peptide G3 possesses stage-specific activity, initially preventing the adhesion and colonization of *Streptococcus mutans*, and subsequently targeting extracellular DNA to interfere with mature biofilm structures (Zhang et al., 2020). Likewise, Cec4 shows both inhibitory and eradicative effects on *Klebsiella pneumoniae* biofilms and can eliminate biofilms generated by CRAB, albeit at increasingly high concentrations (256–512 μg/mL) (Peng et al., 2023; Li et al., 2025). Nevertheless, AMPs that combine biofilm inhibition and eradication functions remain scarce. To solve the limitations of single AMPs in biofilm eradication, researchers have explored innovative strategies. For instance, KKd-11 can self-assemble into hydrogels, facilitating dual intervention through extended proteolytic degradation resistance and prolonged antimicrobial activity (Guo et al., 2021). In another approach, the combined application of melimine or Mel4 with ciprofloxacin has shown synergistic effects in eradicating *Pseudomonas aeruginosa* biofilms (Yasir et al., 2020). Unfortunately, our study found that AaeAP2a did not exhibit a significant eradicative effect on mature *Acinetobacter baumannii* biofilms at 4 × MIC (data not shown). Future studies may use combined strategies such as AMP + antibiotics, AMP + quorum sensing inhibitors, or nanocarrier-based delivery systems to explore AaeAP2a biofilm eradication (Abdelhamid and Yousef, 2023; Liu et al., 2025).

After 20 generations of sub-MIC exposure, AaeAP2a exhibited a remarkably low resistance induction rate (Figure 3A), which is consistent with the low abundance and limited transmission of resistance genes (Chen et al., 2023), the multiple antimicrobial mechanisms (Bucataru and Ciobanasu, 2024), as well as rapid bactericidal effect of AMPs (Zhang et al., 2022). Safety and stability are essential prerequisites for the clinical application of pharmaceutical agents. Assessments of hemolytic activity and cytotoxicity have preliminarily confirmed that AaeAP2a exhibits promising biosafety *in vitro* (Figures 4A,B). However, to truly advance its clinical translation, more comprehensive safety data are needed, including long-term toxicity, toxicokinetics, and tissue compatibility. Notably, the hemolytic activity of AaeAP2a in our study is considerably lower than previously reported. Du et al. observed complete lysis of a 2% erythrocyte suspension at 64 μg/mL, whereas our experiments showed that even at 200 μg/mL (with a 4% suspension), the hemolysis rate remained below 5%. This discrepancy may be attributed to differences in red blood cell sources; our study utilized mouse erythrocytes, in contrast to the horse erythrocytes used by Du et al. The compound also displayed considerable stability, retaining its efficacy after exposure to high temperatures (100 °C), a wide pH range (4–10), and multiple freeze–thaw cycles. Notably, its activity was diminished only at elevated concentrations of divalent cations (Ca^2+^, Mg^2+^ > 50 mM) or in the presence of trypsin (> 40 μg/mL), reflecting a competitive displacement effect of Mg^2+^/Ca^2+^ ions on lipopolysaccharides (LPS), as previously reported (Sun et al., 2025). Furthermore, its exceptional thermal and freeze–thaw stability, combined with robust long-term storage stability at ambient temperature, underscores its potential for formulation development. Nevertheless, strategies such as altering administration routes (e.g., intravenous, inhalation, or topical), enhancing intrinsic stability (e.g., through D-amino acid substitution, terminal closure, cyclization, or polymerization), and optimizing delivery systems (e.g., liposomes, nanoparticles, or hydrogels) may further mitigate the identified vulnerabilities (Zheng et al., 2025).

Given that AaeAP2a does not readily induce bacterial resistance, we hypothesized that it can offer distinct advantages over the single-target mechanism typical of conventional antimicrobial agents. Indeed, previous studies have demonstrated that AMPs can exert broad-spectrum bactericidal effects through a multimodal synergistic mechanism (Xuan et al., 2023). Numerous AMPs, such as the modified peptide 2K4L (Ji et al., 2023) and the novel peptide LL-1 (Zhou L. et al., 2022) derived from the Linnaeus toadstool, achieve sterilization by disrupting cell membranes. These peptides markedly increase the permeability of MDRAB and *E. coli* cell membranes, leading to intracellular leakage and cell death. Similarly, our team members Zhang et al. (2015), Wang et al. (2019), and Wang et al. (2019) demonstrated that the antimicrobial peptide JH-3 inhibits *Salmonella* and *E. coli* by disrupting the integrity of their cell walls and membranes. In this study, we find that AaeAP2a significantly enhances the permeability of the outer membrane in *A. baumannii* while compromising the integrity of its inner membrane (Figures 5, 6). Moreover, AaeAP2a disrupts the bacterial membrane potential, accompanied by a disciplinary change of ΔpH, and subsequently perturbs key bacterial metabolic processes. This perturbation resulting in the decomposition of the electron transport chain, ATP loss, ROS, and •OH formation (Figure 7). These findings corroborate previous studies indicating that AMPs can provoke membrane destabilization, PMF collapse, energy metabolism impairment, and oxidative stress (Xia et al., 2021; Shi et al., 2022). This multi-target synergistic bactericidal mechanism successfully overcomes the limitations of conventional antibiotics and provides a persuasive medical justification for AaeAP2a’s potent antibiotic effects.

In conclusion, we conducted a thorough investigation of AaeAP2a, which shows strong *in vitro* bactericidal activity and effective *in vivo* therapeutic potential against CRAB. AaeAP2a also demonstrates favorable biosafety and stability. Mechanistic studies indicate that its mode of action involves increased permeability of both inner and outer bacterial membranes, membrane depolarization and potential dissipation, impaired ATP synthesis, and oxidative damage. These findings position AaeAP2a as a promising candidate in the fight against antibiotic resistance. Nonetheless, the crystal structure, in vivo half-life, and metabolic pathway of AaeAP2a remain to be fully elucidated. Further research is warranted to address translational challenges through yield optimization and cost reduction.

## Data Availability

The original contributions presented in the study are included in the article/supplementary material, further inquiries can be directed to the corresponding authors.
